# Contrast Water Therapy and Exercise Induced Muscle Damage: A Systematic Review and Meta-Analysis

**DOI:** 10.1371/journal.pone.0062356

**Published:** 2013-04-23

**Authors:** François Bieuzen, Chris M. Bleakley, Joseph Thomas Costello

**Affiliations:** 1 Laboratory of Sport, Expertise and Performance, Institut National du Sport, de l’Expertise et de la Performance (INSEP), Paris, France; 2 Ulster Sports Academy, Faculty of Life and Health Sciences, University of Ulster, Newtownabbey, County Antrim, Northern Ireland; 3 Institute of Health and Biomedical Innovation, Queensland University of Technology, Kelvin Grove, Queensland, Australia; 4 Department of Physical Education and Sport Sciences, University of Limerick, Castletroy, Limerick, Ireland; The University of Queensland, Australia

## Abstract

The aim of this systematic review was to examine the effect of Contrast Water Therapy (CWT) on recovery following exercise induced muscle damage. Controlled trials were identified from computerized literature searching and citation tracking performed up to February 2013. Eighteen trials met the inclusion criteria; all had a high risk of bias. Pooled data from 13 studies showed that CWT resulted in significantly greater improvements in muscle soreness at the five follow-up time points (<6, 24, 48, 72 and 96 hours) in comparison to passive recovery. Pooled data also showed that CWT significantly reduced muscle strength loss at each follow-up time (<6, 24, 48, 72 and 96 hours) in comparison to passive recovery. Despite comparing CWT to a large number of other recovery interventions, including cold water immersion, warm water immersion, compression, active recovery and stretching, there was little evidence for a superior treatment intervention. The current evidence base shows that CWT is superior to using passive recovery or rest after exercise; the magnitudes of these effects may be most relevant to an elite sporting population. There seems to be little difference in recovery outcome between CWT and other popular recovery interventions.

## Introduction

Various modalities of recovery are currently used by athletes in an attempt to offset the negative effects of strenuous exercise. Elite-level athletic participation necessitates recovery from many physiological stressors [1], [2], including fatigue to the musculoskeletal, nervous and metabolic systems [3]. Athletic participation may also cause exercise induced muscle damage (EIMD), which may lead to delayed onset muscle soreness (DOMS) [3].

EIMD frequently occurs after unaccustomed exercise, particularly if the exercise involves a large amount of eccentric (muscle lengthening) contractions [2], [4]–[10]. This phenomenon was first reported in the literature in the early 1900’s [11] and research in the area has increased in recent decades as elite athletes seek to enhance their training, recovery and subsequent performance. Although, the exact mechanisms responsible for damage, repair and adaptation have not been delineated, early research has suggested that the initial disruption to skeletal muscle following exercise is attributed to progressive degeneration of certain myofibres [12]. This is followed by secondary damage potentially initiated by a disruption to the intracellular Ca^2+^ homeostasis [5]. However, according to Cheung et al. [2] and colleagues as many as six theories have been proposed as potential aetiological explanations for this muscular pathology. Purely eccentric contractions are not the only causes of EIMD. ‘High-intensity exercises’ leading to repeated eccentric muscle contractions [13], tissue vibrations [14], high levels of collisions or impacts [15] and involving a high metabolic cost have also been identified as a physiological [16] and mechanical stress leading to EIMD.

The symptoms of EIMD manifest as a temporary reduction in muscle force [17]–[19], disturbed joint position sense [20]–[22] and reduced athletic performance [23], [24]. Furthermore, EIMD increases inflammatory markers both within the injured muscle and in the blood [25], [26] as well as increasing muscle soreness, stiffness and swelling [19], [27], [28]. The intensity of discomfort and soreness associated with EIMD increases within the first 24 hours, peaks between 24 and 72 hours, before subsiding and eventually disappearing 5–7 days after the exercise [5], [27].

In an attempt to alleviate the symptoms of EIMD several methods of cryotherapy such as ice massage [28], [29], cold water immersion [1], [30], [31], Whole Body Cryotherapy chambers [32]–[34], and other therapeutic techniques including hyperbaric oxygen therapy, non-steroidal anti-inflammatory drugs, compression garments, stretching, electromyostimulation, combination modalities, homeopathy, ultrasound and electrical current modalities are being used by athletes [3]. Cryotherapy is proposed to help recovery from EIMD, and subsequent muscle soreness, by altering tissue temperature and blood flow [32]. Furthermore, the compressive effect of water immersion is thought to create a displacement of fluids from the periphery to the central cavity [35]. This hydrostatic pressure results in multiple physiological changes, including an increase in substrate transport and cardiac output as well as a reduction in peripheral resistance and extracellular fluid volume via intracellular-intravascular osmotic gradients [36]. Cold water immersion is perhaps the most popular method of cryotherapy. Two recent meta-analyses [1], [3] found low quality empirical evidence that CWI was an effective strategy to reduce DOMS following a range of exercise types, yet its effects on muscle function was less clear.

Contrast Water Therapy (CWT), alternating cold and warm water immersion, is also been offered to athletes as an alternative to cryotherapy and is commonly used within the sporting community [37]–[39]. It has been suggested that CWT may reduce oedema by alternating peripheral vasoconstriction and vasodilation [40]. This theory is commonly referred to as a “pumping action” within the literature. Other physiological effects of CWT that may assist athletic recovery include alterations in tissue temperature and blood flow; reduced muscle spasm and inflammation; and improved range of motion [41], [42]. However, the exact mechanisms by which CWT may improve athletic recovery have yet to be established and presently there is little evidenced-based consensus.

A systematic review of the research findings will help determine the effectiveness of CWT following EIMD. To align with the practical application of CWT therapy, we will focus on ‘eccentric exercise’ and ‘high-intensity exercise’ providing that the physiological stress is enough to affect the symptoms of EIMD. Our aim was to systematically review the literature addressing the effects of CWT, following exercise inducing muscle damage (eccentric exercise and high-intensity exercise), on outcomes relating to DOMS, muscle damage, inflammation, muscle strength and power and to discuss their relevance to the sporting community.

## Methods

### 1. Literature Search Strategy

**Figure 1:**
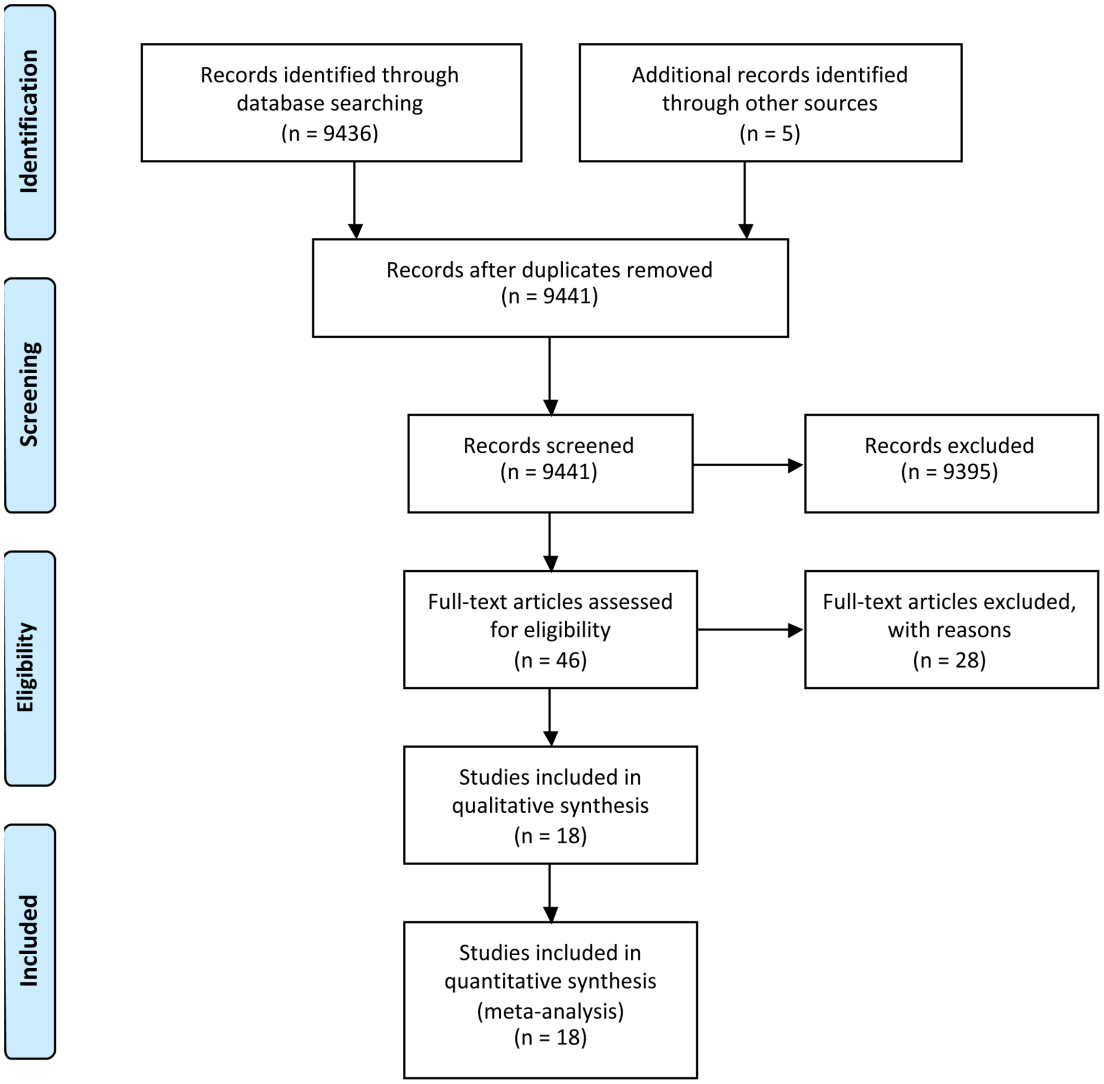
Summary of search strategy and selection process based on included and excluded studies.

The systematic review with meta-analysis was completed in accordance with the recommendations outlined in the Preferred Reporting Items for Systematic Reviews and Meta-Analyses statement [43]. A computerized literature search was conducted, ending in February 2013, using Medline (PubMed), SportDiscus, ProQuest and ISI Web of Knowledge. The following key phrases and their combinations were used: *contrast water therapy*, *muscle soreness, delayed onset of muscle soreness, contrast water immersion, contrast baths, contrast-bath, alternate bath*, *recovery strategy, recovery modality, recovery* and *fatigue*. Reference lists of all articles were examined for identification of further eligible studies (Figure 1).

### 2. Study Inclusion and Exclusion Criteria

Studies must have involved human participants treated with a CWT intervention after exercise. CWT was defined as alternating immersions in hot and cold water. Cold water immersion and hot water immersion are defined as immersion in water temperatures of ≤15°C [3] and >35°C respectively [36], [44]. Studies meeting the following criteria were considered for review: 1) the study design was randomized into an intervention group (CWT) and a control group; 2) a least one outcome measure of muscle soreness, muscle damage, inflammation, muscle strength or power were reported; 3) only outcome variables measured immediately (0–6 h, i.e. <6 h) after the first recovery session and at 24 h, 48 h, 72 h or 96 h post exercise were included; 4) CWT was applied within 1 h post exercise (studies who repeated the CWT protocol on subsequent days were included) and 5) participants could be male or female and of any athletic training status. There were no restrictions placed on the type of exercise or control groups used. Studies using multiple recovery modalities, including CWT in conjunction with another recovery modality, following EIMD were not considered.

### 3. Selection of Studies

Two authors (FB, JC) independently selected trials for inclusion. The titles and abstracts of publications obtained by the search strategy were screened. All trials classified as relevant by either of the authors were retrieved. Based on the information within the full reports, we used a standardized form to select the trials eligible for inclusion in the review. Disagreement between the authors was resolved by consensus, or third party adjudication (CB).

### 4. Data Extraction and Management

Data were extracted independently by two review authors (FB, JC) using a customized form. This was used to extract relevant data on methodological design, eligibility criteria, interventions (including detailed characteristics of the CWT protocols), comparisons and outcome measures. Any disagreement was resolved by consensus, or third-party adjudication (CB). To perform intent-to-treat analysis, where possible, data were extracted according to the original allocation groups, and losses to follow-up were noted. There was no blinding to study author, institution or journal at this stage.

### 5. Measures of Treatment Effect

For each study, mean differences and 95% confidence intervals were calculated for continuous outcomes. For continuous outcomes that were pooled on different scales, standardised mean differences were used. We had planned to preferentially extract data based on changes from baseline (mean change scores); however, the majority of studies reported follow-up scores. In the event that there was no evidence of heterogeneity of effect (P>0.1), a fixed-effect model was used for meta-analysis. In cases where there was evidence of statistical heterogeneity, we checked the results using a random-effects mode.

### 6. Risk of Bias

For all included studies, methodological quality was assessed by two authors independently (FB, CB), using the Cochrane risk-of-bias tool [45]. Each study was graded for the following domains; sequence generation, allocation concealment, blinding (assessor), incomplete outcome data and other sources of bias. For each study, the domains were described as reported in the published study report (or if appropriate based on information from related protocols, published comments or through personal correspondence with the original investigators) and judged by the review authors as to their risk of bias. They were assigned ‘low’ if criteria for a low risk of bias are met or ‘high’ if criteria for a high risk of bias are met. If insufficient detail of what happened in the study was reported, or if what happened in the study was known, but the risk of bias was unknown, then the risk of bias was deemed ‘unclear’ for that domain. Disagreements between authors regarding the risk of bias for domains were resolved by third part evaluation (JC).

### 7. Subgroup Analysis

We undertook subgroup analysis according to the details of the treatment intervention (hot water temperature), and the type of study (parallel versus crossover). An additional subgroup analysis was planned according to methodological quality (high risk versus low risk of bias) however we were unable to meaningfully subgroup studies into high and low quality.

## Results

### 1. Included Studies

Characteristics of included studies are summarized in table 1. There were 18 eligible studies [35], [37], [38], [44], [46]–[59], comprising a total of 356 healthy participants with unequal distribution of gender (male, n = 301; female, n = 55). The average sample size was 19 with the largest study based on 56 participants [52]. Participants tended to be young and mean ages ranged from 14 [51] to 36 years [56]. All eligible studies were randomized controlled trials (n = 7) parallel group trials, [38], [48], [52], [53], [57]–[59] or crossover trials (n = 11) [35], [37], [44], [46], [47], [49]–[51], [54]–[56]. In crossover studies, the time between each condition ranged from 2 days [55] to 8 months [35].

**Table 1:** Summary of included studies.

Author(s)	Participant cohort (training status, sex (m:f), number, age)	Exercise intervention	Classificationof the exercise	[CWT duration and temperature]×number	Starting/Finishing	Number of Treatment Sessions and frequency	Total CWT duration	Outcome variables and time of measurement post exercise (h)
Dawson et al. [46]	Semi-professional male Australian football players(17∶0); 24.2±2,9	Australian football game	High intensity	[1min at 12°C]**×**4[2 min at 45°C]**×**5	Hot/Hot	1	14 min	DOMS (15 48)Vertical jump (15 48)6-sec sprint peak power (15 48)
Elias et al. [47]	Professional male Australian football players(14∶0); 20.9±3.3	60 min Australian football training	High intensity	[1 min at 38°C]**×**7[1 min at 12°C]**×**7	NR	1	14 min	DOMS (1 24 48)CMJ (24 48)Squat Jump (24 48)
Elias et al. [57]	Professional male Australian football players(24∶0); 20.9±3.3	Australian football game	High intensity	[1 min at 38°C]**×**7[1 min at 12°C]**×**7	NR	1	14 min	DOMS (1 24 48)CMJ (24 48)Squat Jump (24 48)
French et al. [48]	Physically active males(16∶0); 24.1±3.2	6×10 leg extension and flexion +5-sec eccentric	Eccentric	[1 min at 8–10°C]**×**4[3 min at 37–40°C]×3	Cold/Cold	1	13 min	DOMS (0 1 24 48)CK (1 24 48)Mb (1 24 48)5RM Squat (48)CMJ (48)
Gill et al. [38]	Elite male rugby players(28∶0); 25±3	Rugby match	High intensity	[1 min at 8–10°C] ×3[2 min at 40–42°C]×3	NR	1	9 min	CK (0 36 84)
Higgins et al. [58]	U20 male rugby players(24∶0); 19.5±0.8	Simulated rugby game	High intensity	[1 min at 10–12°C]×5[1 min at 38–40°C]×5	NR	1	10 min	DOMS (1 48 72 96 144)CMJ (1 48 72 96 144)
Higgins et al. [59]	U20 male rugby players(24∶0); 19.5±0.8	Simulated rugby game	High intensity	[1 min at 10–12°C]×5[1 min at 38–40°C]×5	NR	1	10 min	DOMS (1 24 48)CMJ (1 24 48)
Ingram et al. [49]	Males with team sport experience(11∶0); 27.5±6.0	Simulated team sport: 4×20 min of intermittent running	High intensity	[2 min at 10°C]×3[2 min at 40°C]×3Transfer time 30-sec	NR	2 (24 h)	24 min	DOMS (0 24 48)CK (24 48)CRP (24 48)Leg extension (48)
King and Duffield [50]	Trained netball females(0∶10); 19.5±1.5	Simulated netball match: 4×15 min of intermittent sprint exercise circuit	High intensity	[1 min at 9.7°C]×5[2 min at 39.1°C]×5Hot = warm shower	Cold/Hot	1	15 min	DOMS (0 24)CMJ (24)
Kinugasa and Kilding [51]	Youth male soccer players(19∶0); 14.3±0.7	Soccer match	High intensity	[1 min at 12°C]×3[2 min at 38°C]×3Hot = warm shower	Cold/Hot	1	9 min	DOMS (0 24)CMJ (24)
Kuligowski et al. [52]	Healthy male and female(28∶28); 21.1±3.0∶20.1±2.1	50 eccentric arm extensions	Eccentric	[3 min at 38.9°C]×6[1 min at 12.8°C]×6	NR	4 (24 h)	96 min	DOMS (24 48 72 96)Isometric Elbow Flexion (24 48 72 96)
Pournot et al. [53]	Elite male athlete(19∶0); 21.5±5.6	20-min intermittent exercise (30-sec drop jump and 30-sec rowing)	High intensity	[1.5 min at 42°C]×5[1.5 min at 10°C]×5	Cold/Hot	1	15 min	DOMS (24)CK (1 24)LDH (1 24)Isometric leg extension (1 24)CMJ (1 24)
Robey et al. [54]	Junior Elite male and female Rowers(4∶2); 18.6±0.6Male and female club rowers(8∶6); 20.2±2.2∶21.1±3.6	Stair-climbing running	Eccentric	[1 min at 12°C]×5[2 min at 40°C]×5Hot = warm shower	NR	3 (24 h)	45 min	DOMS (24 48 72)CK (24 48 72)Isokinetic Leg extension (24 48 72)
Stanley et al. [44]	Endurance trained male cyclists (18∶0); 27±7	60 min cycling high-intensity interval training	High intensity	[1 min at 14.2°C]×4[2 min at 35.5°C]×3	Cold/Cold	1	10 min	DOMS
Vaile et al. [37]	Recreational male and female athletes(4∶9); 26.2±5.8	5×10 eccentric leg press	Eccentric	[1 min at 8–10°C]×5[2 min at 40–42°C]×5	Cold/Hot	1	15 min	DOMS (0 24 48 72)CK (0 24 48 72)Isometric Squat (0 24 48 72)Squat jump (0 24 48 72)
Vaile et al. [35]	Strength trained males(38∶0); NR	5×10 eccentric leg press	Eccentric	[1 min at 15°C]×7[1 min at 38°C]×7	NR	4 (24 h)	56 min	DOMS (24 48 72)CK (0 24 48 72)LDH (24 48 72)Mb (24)IL6 (24)Isometric Leg Extension (24 48 72)Squat Jump (24 48 72)
Versey et al. [55]	Trained male cyclists(11∶0); 32.1±7.6	75 min simulated cycling race	High intensity	[1 min at 14.6°C]×3/6/9[1 min at 38.4°C]×3/6/9	Cold/Hot	1	6 min12 min18 min	DOMS (2)Peak power (2)
Versey et al. [56]	Trained male runners(10∶0); 36.8±9.2	3×100 m +3000 m TT +8×400 m	High intensity	[1 min at 14.6°C]×3/6/9[1 min at 38.4°C]×3/6/9	Cold/Hot	1	6 min12 min18 min	DOMS (0.5 0.75 1 1.5)

Values are means ± SD. CK, creatine kinase. CWT, contrast water therapy. CMJ, counter movement jump. DOMS, delayed onset of muscle soreness. RSA, repeated sprint ability. TT, time trial. LDH, lactate dehydrogenase. IL, interleukin. NR, not reported. Mb, myoglobin.

All studies [35], [37], [38], [44], [46]–[59] incorporated a contrast water group, the duration of immersion per session ranged between 6 [55], [56] and 24 [52] minutes and the number of treatments ranged between 1 [6], [37], [38], [44], [46]–[48], [50], [51], [53], [55]–[59] and 4 [35], [52] interspaced by 24 h. All but one study [53] applied cold water immersion for 1 minute. The duration of immersion in hot water ranged between 1 [35], [47], [55]–[59] and 3 [48], [52] minutes; three studies [50], [51], [54] used warm showering for similar time periods. The temperature of cold water ranged from 8°C [37], [38], [48] to 15°C [35] whereas temperature of hot water ranged between 35.5°C [44] and 45°C [46].

### 2. Detail of Comparisons

The 18 studies were divided in six different groups: CWT vs passive intervention (no CWT or rest) [35], [37], [38], [44], [46]–[59]; CWT vs. cold water immersion [35], [44], [47], [49], [50], [52], [53], [57]–[59]; CWT vs. active recovery [38], [50]; CWT vs. compression garment [38], [48]; CWT vs. warm water immersion [35], [52], [53] and CWT vs. stretching [46], [54]. Fourteen studies [35], [38], [44], [46]–[50], [52]–[54], [57]–[59] used more than one relevant treatment comparison and therefore appear in two or more different sections.

CWT versus passive (no intervention/rest) was the most common comparison, which was made by 18 studies [35], [37], [38], [44], [46]–[59]. Passive intervention was defined as either seated rest [35], [37], [38], [44], [47], [49], [50], [54]–[59] or no intervention [46], [48], [51]–[53].

Ten studies compared CWT to CWI [35], [44], [47], [49], [50], [52], [53], [57]–[59]. Almost 95% of the included studies used a CWI immersion temperature of between 10–15°C [35], [44], [46], [47], [49]–[59], while only one used temperatures of lower than 9°C [50]. In 6 studies [35], [44], [47], [52], [53], [57], treatment involved continuous immersion for between 5 and 24 minutes. The remaining studies [49], [50], [58], [59] undertook CWI in sets where participants got out of the water at pre-determined time points; treatment therefore consisted of two sets of five minute immersions. Three studies undertook additional CWI interventions after completing a single exercise session: Ingram et al. [49] (24 h), Kuligowski et al. [52] (24, 48 and 72 h) and Vaile et al. [35] (24, 48 and 72 h).

Three studies compared CWT to warm water immersion [35], [52], [53] in water between 36 and 38°C. The total duration of warm-water immersion was: 14 [35], 15 [53] and 24 minutes [52]. Two studies [38], [50] compared CWT with an active recovery intervention, which involved 15 minutes of jogging at a predetermined and controlled speed [50] or 7 minutes of low-intensity cycling exercise [38].

Two studies [38], [48] compared CWT with compression therapy. For both studies, participants in the compression group wore full length compression garments for 12 h overnight. Two studies [46], [54] compared CWT with stretching therapy which involved 15 minutes of static stretching; based on 30 seconds stretches which were repeated 2–3 times across several muscle groups and joints. A further two studies [55], [56] specifically compared three different treatment durations of CWT (6, 12 or 18 minutes).

### 3. Details of Outcome

Pain was the most commonly reported outcome. Fifteen studies assessed muscle soreness using a Lickert scale or a visual analogue scale (VAS). A 5 point scale was used in one trial [51], 7 point scale in two trials [46], [54], 10 points or 10 cm VAS were used in 11 trials [35], [37], [44], [47]–[50], [53], [55]–[57] and a 12 cm VAS was used by Kuligowski et al. [52]. The written descriptors used at each end of the scale were specified in all but one of the studies [49]. Four studies specified that pain was measured during a functional movement associated with the exercised body part(s) [35], [52], [55], [56]. Although not explicit, it appears that the remaining studies [37], [38], [44], [46]–[51], [53], [54], [57] assessed muscle soreness at rest. Two studies [58], [59] measured pain on pressure (tenderness) using a hand held algometer device and measured pain levels on a VAS (10 cm).

Eight studies recorded muscle strength; the majority measured isolated body regions (knee extension [49], [53], [54], knee flexion [49], hip flexion [49] and elbow flexion [52] using an isokinetic dynamometer to measure torque (Nm) [53], [54] and a strain-gauge [52] or a cable tensiometer [49] to measure force (kg). Two studies measured the ground force (N) from a force-plate during isometric squat movement [35], [37]. Two studies used strain-gauge to measure peak torque during a sprint cycling performance [46], [55].

Biochemical markers were reported in 7 studies [35], [37], [38], [48], [49], [53], [54]. These markers were divided into two subcategories: biomarkers of inflammation (IL-6 [35]; CRP [49]) and muscle damage (creatine kinase (CK) [35], [37], [38], [48], [49], [53], [54]; lactate dehydrogenase (LDH) [35], [53]; Myoglobin (Mb) [35], [48]).

Twelve studies reported power. In 10 studies, power was assessed by measuring vertical jump performance (centimetres (cm) or flight time (sec)) either with [46], [47] or without [47], [48], [50], [51], [53], [57]–[59] counter movement. Using a specialist device, Vaile et al. [35], [37] measured the power produced in Watts (W) during a weighted squat jump.

### 4. Follow-Up

All studies undertook multiple follow-up observations for each outcome. Fifteen studies [35], [37], [38], [44], [47]–[51], [53], [55]–[59] reported multiple follow-ups in the initial stages of the experiment (<6 h) (e.g. pre-exercise, post-exercise, pre-intervention and post-intervention); we focused on outcomes reported immediately after the completion of the recovery intervention and subsequent days. The majority of studies undertook additional follow-ups at 15 [46], 24 [50], [51], [53], [57], [59], 36 [38], 48 [35], [37], [46]–[49], [52], [54], [57]–[59], 72 [35], [37], [52], [54], [58], 84 [38], 96 [52], [58] or 144 [58] hours.

### 5. Risk of Bias

**Figure 2:**
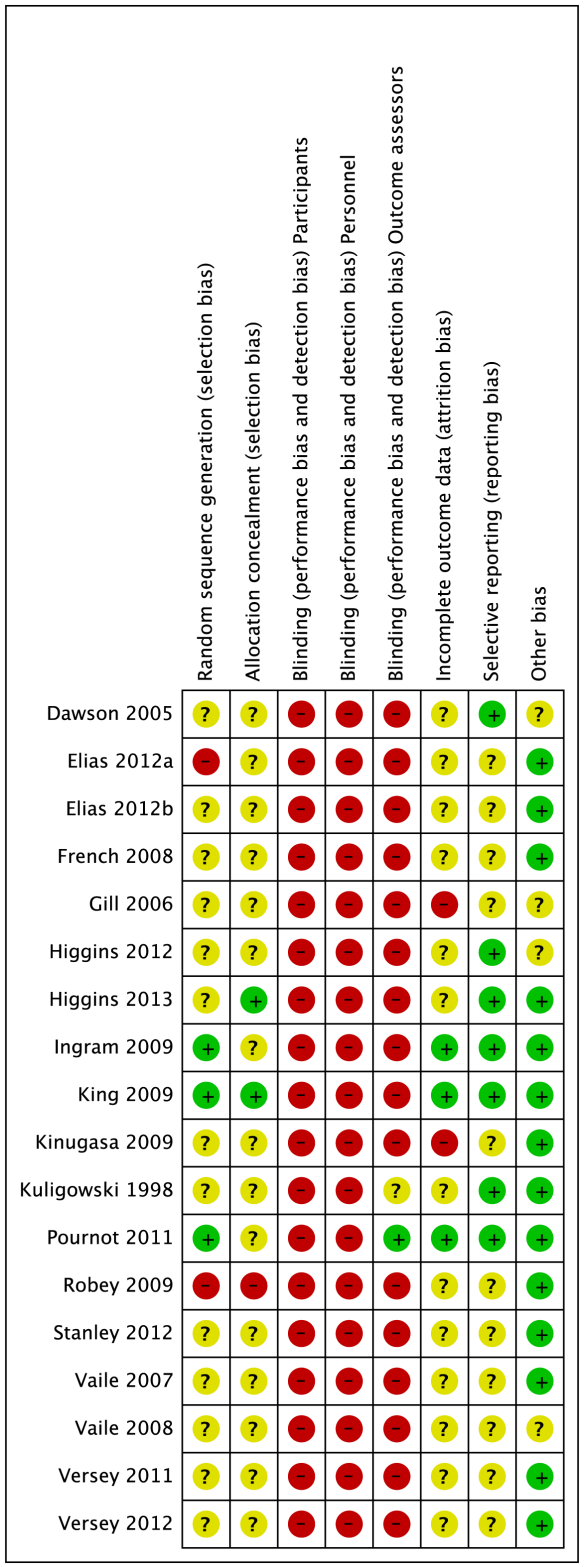
Risk of bias graph: review authors’ judgements about each risk of bias item presented as percentages across all included studies.

**Figure 3:**
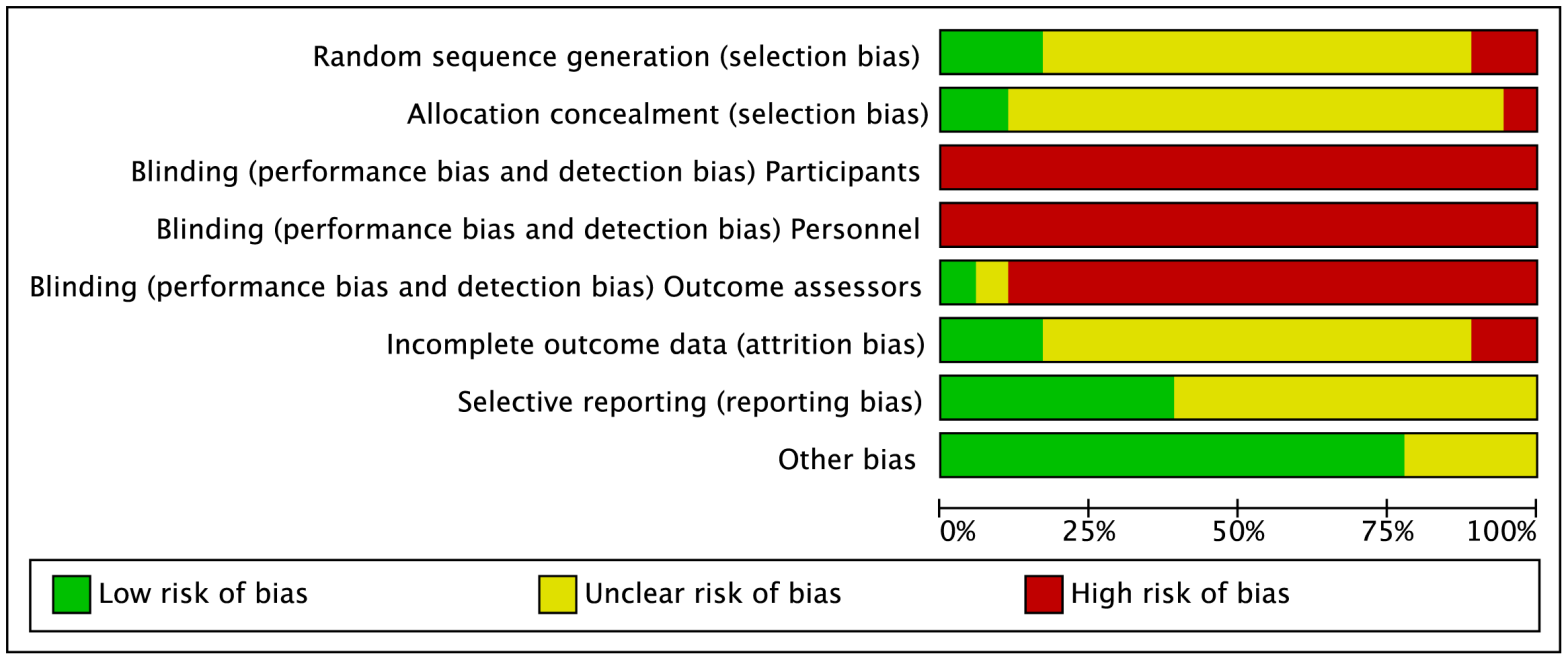
Risk of bias summary: review authors’ judgements about each risk of bias item for each included study.

Full details of the quality assessment are given in figure 2 and 3. Randomisation procedure was described in just three studies [49], [50], [53]; these were clarified after personal correspondence and were based on a computer generated sequence [49] or hat draw [50], [53]. Only two studies [50], [59] carried out adequate allocation concealment, again this was judged following personal correspondence. In the remaining studies, there was no clear indication that the investigators would be unable to predict the prospective group, or in the case of crossovers, the order of treatments to which participants would be allocated (Figure 2; Allocation concealment). None of the studies utilised blinding of participants or care givers and only one study [53] used blinded outcome assessment.

Reporting on incomplete outcome data was poorly described. This item represents the attrition bias due to amount, nature or handling of incomplete outcome data. After correspondence, three groups of authors [49], [50], [53] confirmed no losses to follow-up or violation from the study protocol. The remainder of studies [35], [37], [38], [44], [46]–[48], [51], [52], [54]–[59] provided no information on drop outs, exclusion, missing data or approach to analysis.

None of the studies made any reference to a published protocol however seven studies [46], [49], [50], [52], [53] {Higgins, 2013 #122048; Higgins, 2012 #122884} clearly described outcomes and follow-up times with corresponding results presented by intervention group. In 4 cases [35], [38], [53], [54], additional group summary data were provided by corresponding authors in order to calculate effect size. Data were extracted from graphs in 2 studies [37], [59].

All studies provided in-depth descriptions of the exercise protocols based on exercise type, duration, and intensity. All but two studies [35], [38] provided adequate detail on co-interventions that were used across intervention groups.

### 6. Contrast Water Therapy versus Passive (No Intervention/Rest)

#### 6.1. Muscle soreness

**Figure 4:**
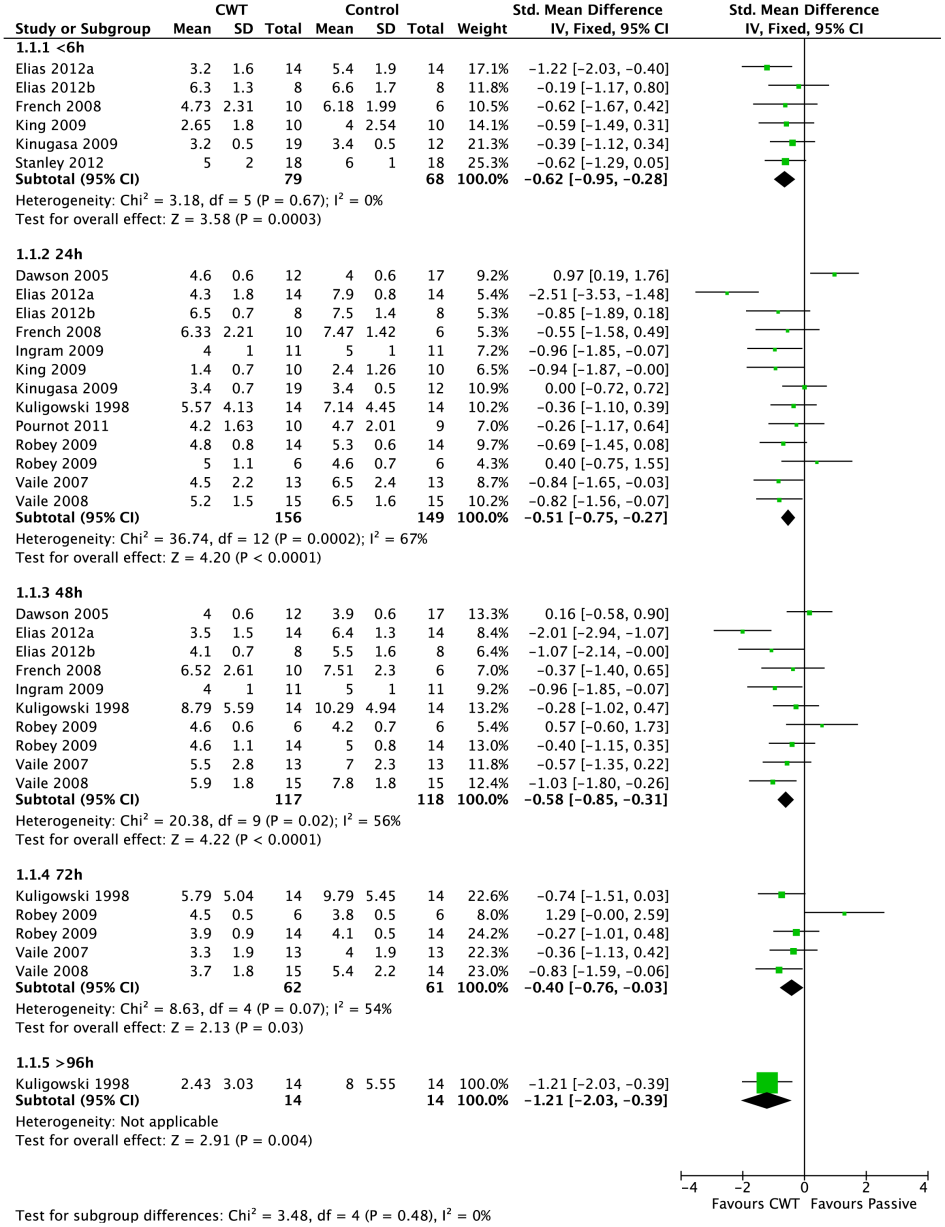
Forest plot of comparison: Contrast vs. Passive, outcome: Muscle soreness: various scales Likert and VAS.

*Pain (muscle soreness: VAS, various scales or scores; highest values = worst pain)* - Thirteen studies [35], [37], [44], [46]–[54], [57] in this comparison presented data on muscle soreness based on various analogue scores or scales. Pooled results are presented in five subcategories based on follow-up time (Figure 4). At all follow-up times, pooled results showed significantly lower levels of muscle soreness in the CWT group (<6 h: SMD −0.62, 95% CI −0.95 to −0.28, 6 trials); (24 h: SMD −0.51, 95% CI −0.75 to −0.27, 13 trials); (48 h: SMD −0.58, 95% CI −0.85 to −0.31, 10 trials); (72 h: SMD −0.40, 95% CI −0.76 to −0.03, 5 trials); (>96 h: SMD −1.21, 95% CI −2.03 to −0.39, 1 trial). However, there was significant heterogeneity in two analyses (24 and 48 hours). While increasing the 95% confidence intervals, the findings in favour of CWT were upheld when applying the random-effects model for all but one follow up time (72 hours). In the 24 and 48 hours analyses, crossover trials have been combined with parallel group trials. Subgroup analysis by study design showed no statistically significant differences between the pooled results of cross-over trials and parallel group trials at both follow-up times. For 24 hours, test for subgroup differences: Chi^2^ = 2.14, df = 1 (P = 0.14), I^2^ = 53.4%); and for 48 hours, test for subgroup differences: Chi^2^ = 1.84, df = 1 (P = 0.17), I^2^ = 45.8%). This seems to suggest that the findings in favour of CWT are not dependent on study design (i.e. crossover or parallel group trials).

We also performed subgroup analyses according to the temperature of the hot water immersion component (> or <40°C). This subgroup analysis showed significant difference only at 48 hours when applying the random-effects model (48 hours: test for subgroup differences: Chi^2^ = 10.93, df = 1 (P<0.001), I^2^ = 90.9%). These suggest that effects were stronger when the temperature of hot water immersion component was less than 40°C [35], [47], [48], [52], [57] rather than more than 40°C [37], [46], [49], [54]. However, it should be noted that there was heterogeneity within each subgroup.

*Pain (Tenderness: algometer)* - The two studies [58], [59] reporting this outcome found no differences between groups for all but one follow up time (24 hours).

#### 6.2. Muscle damage

**Figure 5:**
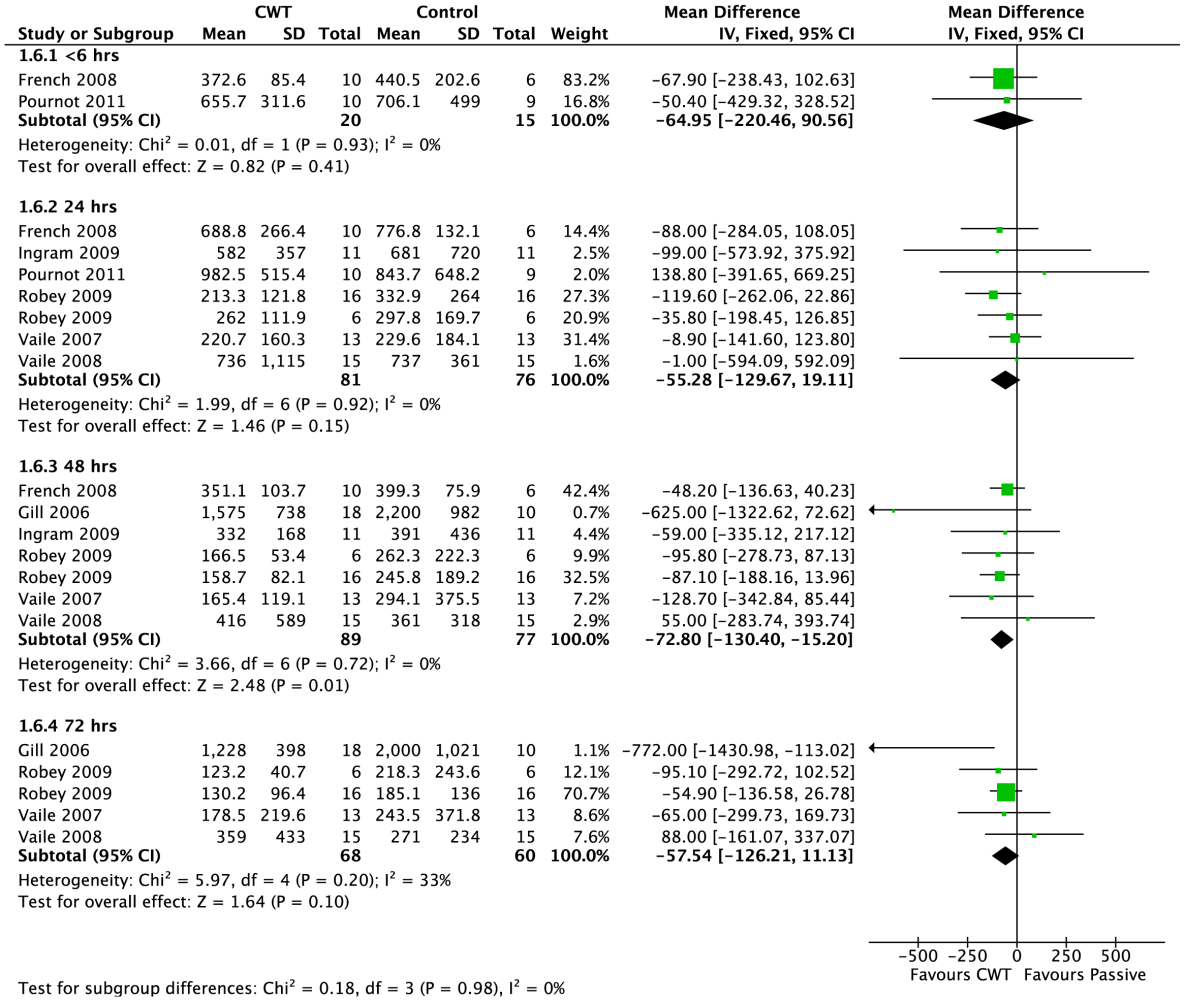
Forest plot of comparison: Contrast vs. Passive, outcome: Muscle damage (CK).

Creatine kinase (CK) was the most commonly reported biomarker. Pooled results are presented in five subcategories based on follow-up time (Figure 5). There were no significant differences between groups at <6 hours (MD −64.95 U.L^−1^, 95% CI −220.46 to 90.56, 2 trials) and 24 hours (MD −55.28 U.L^−1^, 95% CI −129.67 to 19.11, 7 trials). At 48 and 72 h follow ups, pooled results show significantly lower level of creatine kinase in contrast water immersion group (48 h: MD −72.80 U.L^−1^, 95% CI −130.4 to −15.2, 7 trials); (72 h: MD −57.54 U.L^−1^, 95% CI −126.21 to 11.13, 5 trials).

Two studies [35], [53] recorded lactate dehydrogenase (LDH) levels. At <6 h, Pournot et al. [53] found significantly lower levels of LDH within the passive group (MD 108.0 U.L^−1^ vs CWT group, 95% CI 3.11 to 212.9, 1 trial). Pooled results from two studies [35], [53] found similar trends at 24 h follow up (MD 61.24 U.L^−1^ [95% CI −44.46 to 166.94, 2 trials, random effects modelling] however this was not statistically significant. At 48 and 72 hours, Vaile et al. [35] showed no significant difference between groups.

There were no between group differences in myoglobin at <6 hours [48] or at 24 hours [35], [48]. French et al. [48] reported a significant lower level of myoglobin in contrast water immersion group at 48 h (MD −2.70 µg.L^−1^, 95% CI −4.17 to −1.22, 1 trial).

#### 6.3. Inflammation

Only two studies reported on inflammatory markers. There were no statistically significant differences between groups in terms of C-reactive protein (CRP) at 24 h or 48 h [49] or interleukine-6 (IL-6) at 24 hours [35].

#### 6.4. Muscle strength (change from baseline)

**Figure 6:**
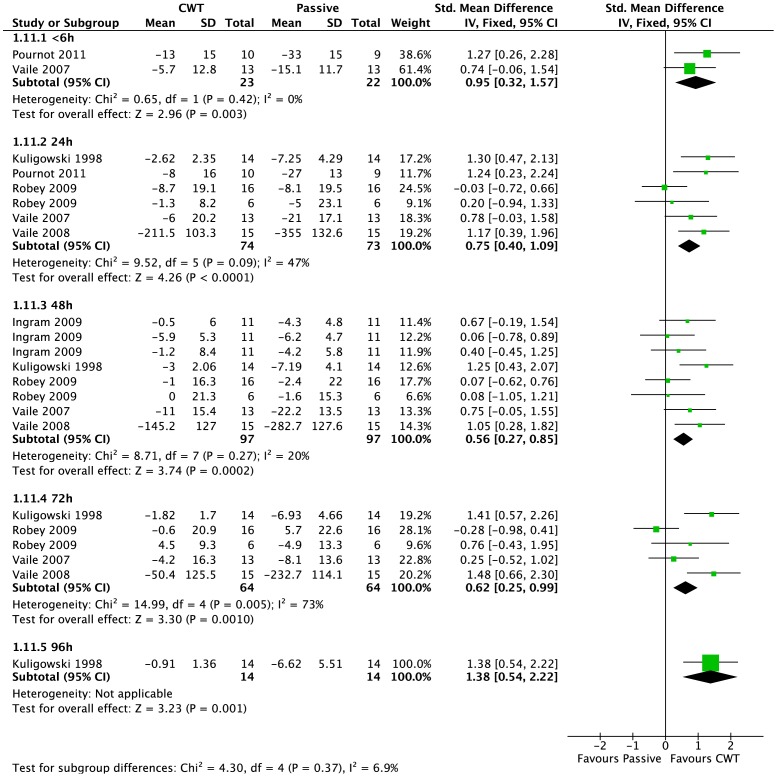
Forest plot of comparison: Contrast vs. Passive, outcome: Muscle Strength (Change from baseline).

Six studies [35], [37], [49], [52]–[54] reported maximal strength at various follow-up times, based on the change from baseline value (this data was extracted directly from the manuscript or calculated after personal correspondence) (Figure 6). Pooled results showed significantly lower changes from baseline in the contrast water immersion group at all follow-up time points (<6 hours: SMD 0.95, 95% CI 0.32 to 1.57, 2 trials; 24 hours: SMD 0.75, 95% CI 0.40 to 1.09, 6 trials; 48 hours: SMD 0.56, 95% CI 0.27 to 0.85, 8 trials; 72 hours: SMD 0.62, 95% CI 0.25 to 0.99, 5 trials; 96 hours: SMD 1.38, 95% CI 0.54 to 2.22, 1 trials). We noted heterogeneity within some of these analyses (72 h); however findings in favour of contrast water immersion were upheld at all follow-ups except at 72 hours (SMD 0.70, 95% CI −0.03 to 1.43, 5 trials) using a random-effects model.

#### 6.5. Muscle power (follow up score and change from baseline)

Muscle power based on jump height was reported by nine studies from their follow-up scores [35], [37], [46], [47], [51], [53], [57]–[59] over a range of time points post intervention. Except at <6 h, pooled results from these studies had low heterogeneity at all follow-up time points (<6 hours: Chi^2^ = 15.31, df = 2 (P<0.001), I^2^ = 87%; 24 hours: Chi^2^ = 10.01, df = 7 (P = 0.19), I^2^ = 30%; 48 hours: Chi^2^ = 5.72, df = 6 (P = 0.46), I^2^ = 0%; 72 hours: Chi^2^ = 1.19, df = 2 (P = 0.55), I^2^ = 0%). There were no differences between groups when applying the random-effects model (<6 hours: SMD 0.47, 95% CI −1.28 to 2.23, 3 trials; 24 hours: SMD 0.12, 95% CI −0.23 to 0.47, 8 trials; 48 hours: SMD 0.19, 95% CI −0.13 to 0.51, 7 trials; 72 hours: SMD 0.09, 95% CI −0.38 to 0.55, 3 trials; >96 hours: SMD −0.43, 95% CI −1.43 to 0.56, 1 trials).

The use of the change from baseline score was only reported in three studies [33], [35], [50]. However, in contrast to results coming from follow-up scores, pooled results showed a significantly lower muscle power loss with CWT at 24 (SMD 0.62, 95% CI 0.13 to 1.11), 48 (SMD 1.39, 95% CI 0.58 to 2.20) and 72 (SMD 1.54, 95% CI 0.71 to 2.37) hours follow-up time points.

### 7. Contrast Water Therapy versus Cold Water Immersion

#### 7.1. Muscle soreness

*Pain (muscle soreness: VAS, various scales or scores; highest values = worst pain)* – Height studies [35], [44], [47], [49], [50], [52], [53], [57] compared muscle soreness after contrast and cold water immersion. No statistical between treatment differences were observed in pooled data when applying the random-effects model at <6 h (SMD 0.41, 95% CI −0.34 to 1.16, 3 studies), 24 h (SMD 0.32, 95% CI −0.28 to 0.92, 7 studies), 48 h (SMD 0.38, 95% CI −0.38 to 1.14, 5 studies), 72 h (SMD −0.15, 95% CI −0.68 to 0.38, 2 studies) and 96 h (SMD 0.12, 95% CI −0.62 to 0.86, 1 studies).

*Pain (Tenderness: algometer)* – The two studies [58], [59] reporting this outcome found no differences between groups for all but one follow up time (48 hours).

#### 7.2. Muscle damage

There were trends towards lower levels of CK in the cold water immersion group in comparison to contrast water immersion, at all time points (<6 h: MD 68.70 U.L^−1^, 95% CI −217.95 to 355.35, 1 trial; 24 hours: MD 204.98 U.L^−1^, 95% CI −12.11 to 422.07, 3 trials; 48 hours: MD 35.62 U.L^−1^, 95% CI −110.46 to 181.69, 2 trials; 72 hours: MD 155.00 U.L^−1^, 95% CI −137.71 to 447.71, 1 trial) however these did not reach statistical significance. There were no between group differences for lactate dehydrogenase marker at <6 hours and 24 hours follow-ups. Results from a single study [35] reported significant differences in favour of cold water immersion at 48 and 72 hours [35]. One trial [35] found no significant difference between groups in the level of myoglobin marker at 24 hours.

#### 7.3. Inflammation

Two trials [35], [49] found no significant differences between groups in inflammatory markers (CRP and IL-6) at 24 and 48 hours follow-ups.

#### 7.4. Muscle strength (change from baseline)

Four studies [35], [49], [52], [53] reported strength. Only two studies [49], [52] reported a significant between group difference; this was in favour of contrast water immersion at 48 hours only for Ingram et al. [49] study and for all but one follow-up times (24 hour) for Kuligowski et al. [52].

#### 7.5. Muscle power (follow up score)

Seven studies [35], [47], [50], [53], [57]–[59] reported muscle power. Again the details of the measuring device, and joint movements tested were different across studies. There were no significant differences between groups at any of the five follow-up times.

### 8. Contrast Water Therapy versus Warm Water Immersion

#### 8.1. Muscle soreness

Three studies made this comparison [35], [52], [53]. At 24 and 96 hours follow-ups, pooled results showed significantly lower levels of muscle soreness in the CWT group at 24 h (SMD −0.73, 95% CI −1.21 to −0.25, 3 trials); and 96 h (SMD −0.97, 95% CI −1.76 to −0.18, 1 trial). There were also trends towards lower levels of soreness in this group at the remaining follow up points, however these were not statistically significant (48 h: SMD −0.19, 95% CI −0.73 to 0.34, 2 trials); (72 h: SMD −0.42, 95% CI −0.97 to 0.12, 2 trials).

#### 8.2. Muscle damage

There were trends from two studies [35], [53] that warm water immersion was associated with lower levels of muscle damage at 24, 48 and 72 hours follow-ups; this was consistent for all three biomarkers (CK, LDH and Mb), but the differences between groups were not statistically significant.

#### 8.3. Muscle strength (change from baseline)

Maximal strength was reported by three studies [35], [52], [53] over a range of time points post intervention. At <6 hours follow-up time point, pooled results tended to favour the CWT group but showed no significant differences. At all four subsequent times, pooled results show significantly lower changes from baseline in the CWT group (24 hours: SMD 0.70, 95% CI 0.21 to 1.18, 3 trials; 48 hours: SMD 0.70, 95% CI 0.14 to 1.26, 2 trials; 72 hours: SMD 0.58, 95% CI 0.03 to 1.14, 2 trials; 96 hours: SMD 0.77, 95% CI 0.00 to 1.55, 1 trial). We noted moderate to high levels of heterogeneity within these analyses; findings in favour of CWT were upheld at all follow-ups except at 72 hours (SMD 0.59 [−0.21, 1.38], 2 trials]) using a random-effects model.

#### 8.4. Muscle power

Pournot et al. [53] and Vaile et al. [35] found a significant difference between groups in favour of warm water immersion in vertical jumping performance at <6 (SMD −1.13 [−2.12, −0.14]) and 72 hours respectively (SMD −0.95 [−1.77, −0.12]. There were no significant differences between groups at 24 hours (SMD −0.45 [−2.05, 1.15], 2 studies) and 48 hours (SMD 0.44 [−0.34, 1.23]).

### 9. Contrast Water Therapy versus Active

King and Duffield [50], in a cross-over trial involving 10 netballers, found a trend to lower levels of muscle soreness in favour of CWT at 24 hours (MD −0.80 (10 cm scale), 95% CI −1.83 to 0.23), the difference between the two groups was not statistically significant. They found no difference between groups in terms of decrement in power (measured by the percentage decrement in jump height over five repetitions) at 24 hours (MD 0.40%, 95% CI −3.86% to 4.66%). Gill et al. [38] showed no differences levels CK levels at 48 hours, but found significantly lower levels within the active group at 72 hours (MD 262 U.L^−1^, 95% CI 40.16 to 483.84).

### 10. Contrast Water Therapy versus Compression

Only French et al. [48] and Gill et al. [38] compared CWT with compression therapy. After an eccentric exercise protocol, French et al. [48] showed no between group differences at <6, 24 and 48 hours follow-up times for muscle soreness. There were however significant effects at 24 and 48 hours in favour of CWT for CK (24 hours: MD −343.5 U.L^−1^, 95% CI −552.11 to −134.89; 48 hours: MD −200.5 U.L^−1^, 95% CI −292.42 to −108.57) and Mb (24 hours: MD −32.5 µg.L^−1^, 95% CI −45.82 to −19.18; 48 hours: MD −10.90 µg.L^−1^, 95% CI −14.39 to −7.41) biomarkers. Gill et al. [38] showed no between group difference for CK after a rugby match.

### 11. Contrast Water Therapy versus Stretching

Two studies [46], [54] compared CWT with stretching therapy. There were no between group differences at any follow up time points for any outcomes: muscle soreness, muscle strength, muscle power (vertical jump) and muscle damage (CK).

### 12. Treatment Dose

Two studies [55], [56] compared different dosages of CWT. Both studies compared treatment durations of either 6, 12 or 18 minutes, after either high intensity cycling [55] or running exercise [56]. There were insufficient data to calculate effect sizes, however neither study reported any significant differences between CWT groups in terms of muscle soreness.

### 13. Adverse Effects

There were no adverse effects reported in individual studies. We did however note that there was little evidence within the reviewed trials to suggest that they undertook active surveillance of pre-defined adverse events relating to interventions.

## Discussion

### 1. Quality of Evidence

Overall the study quality in this review was low. The majority of studies had a high risk of bias making the validity of most of the results uncertain. We were unable to meaningfully subgroup studies into high and low quality. The sample size of included studies was also consistently small, raising questions as to the power of individual trials. Previous Cochrane reviews [3], [60], [61] examining recovery interventions after sport and exercise have reported similar limitations.

Eleven studies used crossover designs. Crossover designs can risk certain carry-over effects between treatment periods, which are not present in parallel group designs. The most likely source of carry-over in this area of research is insufficient recovery from the first exercise bout. This carry-over may have been minimised in the current review as the crossover studies generally used trained individuals completing familiar sporting activities such as various football codes, cycling or rowing. We also undertook additional subgroup analysis by study design which showed no statistically significant differences between the pooled results of crossover trials and parallel group trials. Subgroup analyses based on physiological stress (i.e. eccentric exercise or high-intensity exercise) were considered but were not undertaken due to the limited number of studies.

### 2. Contrast Water Therapy versus Passive (No Intervention/Rest)

CWT resulted in significantly greater improvements in muscle soreness recovery compared to no intervention/rest. This was based on pooled data at four time points (<6, 24, 48 and 96 hours), with findings upheld when a random-effects model was applied. It is important to consider the clinical relevance of these findings. The Minimal Important Difference (MID) [62] for pain reduction in musculoskeletal conditions, has been estimated between 14 [63] and 25% [30]; this magnitude of reduction was achieved in some of the included studies at <6 h [47], [48]; 24 h [37], [47], [52]; 48 h [35], [37], [47], [57]; 72 h [35], [52] and 96 h [52] follow-up points. Of note when the results for all the trials were adjusted to fit the same 10 cm VAS, many of the reductions were not clinically relevant (MD in % at <6 h: 8.7%; 24 h: 6.8%; 48 h: 5.7% and 72 h: 0.8%). These minimally important differences should be considered in the design of future studies in this area.

We can only postulate the underpinning mechanisms for reduced muscle soreness with CWT. A common practice in this review was that CWT finished with immersion in cold water. The analgesic effects of cryotherapy are well documented and include decreased nerve conduction velocity and excitability [64] and reduced neural (nociceptive) transmission [36], [65], [66]. In addition, Gregson et al. [42] have recently suggested that blood flow to muscle may be lower after cold application. This may be due to an activation of the thermal nociceptors, leading to a change in sympathetic nerve activity and consequently reduced arterial flow. From this, the physiological effect of cold water is thought to be partially mediated through temperature-induced reductions in microvascular blood flow around the damage site, which in turn reduce oedema and the induction of inflammatory events [67], [68].

Others have suggested that CWT might decrease pain perception by directly influencing inflammatory pathways, cumulating in an attenuation of nociceptor sensitisation [52], exercise induced oedema [69] and leukocyte infiltration [36]. This is difficult to substantiate based on current evidence. We found just two studies [35], [49] examining inflammatory biomarkers (interleukine-6 (IL-6) and C-reactive protein (CRP)), but there were no significant differences between the two recovery modalities. Future studies in this area should ensure that the timing of outcomes directly aligns with the expected peaks of IL-6 and CRP after exercise.

The direction and magnitude of effects in both our primary and secondary outcome measures were comparable to other reviews investigating the effectiveness of cold water immersion after exercise [1], [3]. The nature of water immersion may offer a generic psychological benefit whereby athletes simply feel more ‘awake’ with a reduced sensation of pain and fatigue after exercise [39]. Further research is needed to determine whether CWT offers any additional physiological effect to single immersions in cold water.

#### 2.1. Muscle strength and power

Maximal voluntary force generating capacity may be the most relevant marker of muscle damage [70], [71]. This was a popular outcome measure in the current review. In contrast to muscle power loss, pooled data showed that CWT significantly reduced muscle strength loss at each follow-up time in comparison to a passive recovery. Again we can only speculate the mechanisms for this. In resting subjects, Fiscus et al. [72] observed that CWT is associated with an increase in limb blood flow during warm immersion following by a decrease during cold immersion. This alternate vasodilatation and vasoconstriction of the peripheral blood vessels or “pumping action” has been proposed to increase lactate clearance [39], decrease oedema [37] and increase blood flow [39]. It is also hypothesized that CWT may alter the perfusion of muscle by inducing intracellular-intravascular fluid shift, which might result in an attenuated immune response and reduced the myocellular damage [37].

#### 2.2. Muscle damage

We observed significantly lower values of CK and (Mb) plasma concentrations from 48 hours post exercise with CWT in comparison to passive recovery without any changes on lactate dehydrogenase. Exercise-induced hemoconcentration and/or hemodilution, and alterations of tissue clearance can affect CK concentration in the blood (and presumably [Mb]) making any interpretation complex. As such, the relevance of using blood biomarkers to quantify the severity of EIMD has been questioned [65], [70], [73].

### 3. Contrast Water Therapy versus Cold Water Immersion, Warm Water Immersion, Compression, Active Recovery and Stretching

We observed a large number of additional treatment comparisons, but there were few statistically significant findings. Pooled analyses found no differences in term of muscle soreness between CWT and cold water immersion, active recovery, compression or stretching. However, CWT significantly decreases muscle soreness in comparison to a warm water immersion recovery at 24 and 96 hours follow-ups. There were little between group differences for any other reported outcomes, and it is difficult to highlight a superior treatment intervention. Notwithstanding this, it may be unrealistic for athletes to adopt a single recovery intervention post exercise. Many focus on potential cumulative effects of a recovery package consisting of a number of different treatment approaches. Perhaps future research should focus on determining an optimal combination of recovery interventions, or potentially outcome specific effects.

### 4. Comparison to Other Reviews

To our knowledge this is the first systematic review and meta-analysis that sought to assess the effects of CWT on athletic recovery following exercise. Few reviews have systematically examined the effects of therapeutic modalities on recovery. Bleakley et al. [3] have recently reviewed the benefits of using cold water immersion to prevent and treat muscle soreness after exercise. These authors concluded that there was some evidence that cold-water immersion reduces delayed onset muscle soreness after exercise compared with passive interventions involving rest or no intervention. Leeder et al. [1] undertook a similar meta-analysis on the effects of cold water immersion on recovery after strenuous exercise. Similarly, this review illustrated that CWI was an effective strategy to reduce DOMS following a range of exercise types, yet its effects on muscle function was less clear. Torres et al. [74] also reviewed the use of different therapeutic modalities, including massage, cryotherapy, stretching and low-intensity exercise to treat the signs and symptoms of exercise-induced muscle damage. Massage was the only intervention that had a positive effect on the recovery of muscle soreness and function; however, the magnitude of this effect was small and may not be clinically relevant. All three reviews conclude that there is a the lack of high quality, well reported research in this area and state further research is required in the area to elucidate the potential mechanisms underpinning the effects of the specific recovery strategies.

### 5. Limitations and Future Study

An exhaustive search based on electronic databases and complementary sources was undertaken in the current review and meta-analysis. However, we acknowledge that some research in the grey literature (such as conference proceedings) may have been overlooked. None of the included studies had a registered protocol and bias from selective reporting of results was difficult to ascertain. There were a limited number of outcomes where data was extracted from graphs. Although, this data was extracted independently by two review authors, with disagreement resolved by third party adjudication, it still serves as an estimation of treatment effect.

Future studies should incorporate a randomised controlled parallel group design with adequate sequence generation and allocation concealment; use adequate sample sizes with power to detect expected differences and use CWT immersion protocols based on a defined physiological rationale in accordance with the type of exercise. Although we acknowledge that the use of effective and explicit blinding of outcomes is difficult, researchers should consider using a study design incorporating placebo or sham treatment. Finally full reporting of data and an active surveillance of pre-defined adverse events is required in future CWT research.

There was a limited number of females in the reviewed studies. A significant gender effect in serum CK activity, inflammatory cell infiltration, and activation of protein degradation pathways has previously been reported in the literature [26]. Furthermore, thermal response during immersion depends on the fat composition [75] suggesting that gender specific effects should be investigated.

There was a large difference among studies in terms of the water temperatures used for immersion. The mean temperature for the cold component was 11.1°C [range: 8°-15°C] and 39.3 [range: 35.5°- 45°C] for the warm component. This implies that the range between hot and cold varied from 21.3°C [44] to 32°C [46]. Although, this is likely to affect outcomes (due to the relationship between tissue temperature, regulation of the sympathetic drive and muscle blood flow) we were unable to delineate an optimal temperature gradient. This should be considered in future study.

The majority of studies evaluated pain using a visual analogue scale, ranging from 0 ‘normal’ to 10 ‘extremely sore). This provides a subjective measure of “muscle soreness” which is likely to relate to delayed onset muscle soreness. We must acknowledge that in some of the included studies, muscle soreness may have a more complex aetiology and other causes of post exercise muscle pain may have contributed to the outcome scores presented.

Furthermore, the effectiveness of CWT in treating different magnitudes of muscle damage is poorly understood. Further research assessing the benefits of this treatment following mild to severe muscle damage is warranted.

Although, the current review focused on the use of CWT in healthy people, some evidence suggests that other methods of contrast therapy (including hot/cold pack application) is being used as a method of recovery following strenuous exercise [76]. This area should be systematically examined in future reviews. Finally, we have focused on important outcomes relevant to recovery including; muscle soreness, muscle function, inflammation and muscle damage makers. We acknowledge that other key correlates of athletic performance, such as flexibility and neuromuscular function, are known to be reduced following exercise induced muscle damage. Other reviews may also be required to assess these outcome measures following various therapeutic recovery strategies.

### Conclusion

The current evidence base suggests that CWT is superior to using passive recovery or rest after various forms of exhaustive or damaging exercise. The benefits relate to a reduction in muscle soreness, and improved muscle function due to an attenuation of muscle strength loss and muscle power loss after exercise. The magnitudes of these effects seem to be clinically relevant but may be most applicable to elite sport. There are no data available to determine an optimal method of CWT. Furthermore, there seems to be little difference in recovery outcome when CWT is compared to other popular recovery interventions such as cold water immersion, warm water immersion, compression, active recovery and stretching. These conclusions are not definitive based on poor methodological quality and small sample sizes. High quality, well reported research in this area is required.

### Perspectives

CWT is a post exercise recovery modality commonly employed in the sporting community. This review sought to systematically evaluate the effects of this treatment on athletic recovery. Muscle soreness, muscle damage, strength, and power all appear to recover quicker after CWT compared to no intervention. However, when CWT was compared to other commonly employed recovery modalities little difference was observed. Consequently, athletes and coaches can be advised to choose a recovery modality that is best suited to their individual training schedules, preferences, facilities and equipment.
